# Fatty acid metabolism prognostic signature predicts tumor immune microenvironment and immunotherapy, and identifies tumorigenic role of MOGAT2 in lung adenocarcinoma

**DOI:** 10.3389/fimmu.2024.1456719

**Published:** 2024-10-16

**Authors:** Denggang Fu, Biyu Zhang, Wenyan Fan, Fanfan Zeng, Jueping Feng, Xin Wang

**Affiliations:** ^1^ College of Medicine, Medical University of South Carolina, Charleston, SC, United States; ^2^ Key Laboratory of Green Chemical Engineering Process of Ministry of Education, School of Chemical Engineering and Pharmacy, Wuhan Institute of Technology, Wuhan, China; ^3^ Jiangxi Provincial Key Laboratory of Cell Precision Therapy, School of Basic Medical Sciences, Jiujiang University, Jiujiang, Jiangxi, China; ^4^ Jiangxi Provincial Key Laboratory of Preventive Medicine, School of Public Health, Nanchang University, Nanchang, Jiangxi, China; ^5^ Department of Oncology, Wuhan Fourth Hospital, Wuhan, Hubei, China

**Keywords:** lung adenocarcinoma, fatty acid metabolism, prognosis, immune microenvironment, immunotherapy, MOGAT2

## Abstract

**Background:**

Aberrant fatty acid metabolism (FAM) plays a critical role in the tumorigenesis of human malignancies. However, studies on its impact in lung adenocarcinoma (LUAD) are limited.

**Methods:**

We developed a prognostic signature comprising 10 FAM-related genes (GPR115, SOAT2, CDH17, MOGAT2, COL11A1, TCN1, LGR5, SLC34A2, RHOV, and DKK1) using data from LUAD patients in The Cancer Genome Atlas (TCGA). This signature was validated using six independent LUAD datasets from the Gene Expression Omnibus (GEO). Patients were classified into high- and low-risk groups, and overall survival (OS) was compared by Kaplan-Meier analysis. The signature’s independence as a prognostic indicator was assessed after adjusting for clinicopathological features. Receiver operating characteristic (ROC) analysis validated the signature. Tumor immune microenvironment (TIME) was analyzed using ESTIMATE and multiple deconvolution algorithms. Functional assays, including CCK8, cell cycle, apoptosis, transwell, and wound healing assays, were performed on MOGAT2-silenced H1299 cells using CRISPR/Cas9 technology.

**Results:**

Low-risk group patients exhibited decreased OS. The signature was an independent prognostic indicator and demonstrated strong risk-stratification utility for disease relapse/progression. ROC analysis confirmed the signature’s validity across validation sets. TIME analysis revealed higher infiltration of CD8+ T cells, natural killers, and B cells, and lower tumor purity, stemness index, and tumor mutation burden (TMB) in low-risk patients. These patients also showed elevated T cell receptor richness and diversity, along with reduced immune cell senescence. High-risk patients exhibited enrichment in pathways related to resistance to immune checkpoint blockades, such as DNA repair, hypoxia, epithelial-mesenchymal transition, and the G2M checkpoint. LUAD patients receiving anti-PD-1 treatment had lower risk scores among responders compared to non-responders. MOGAT2 was expressed at higher levels in low-risk LUAD patients. Functional assays revealed that MOGAT2 knockdown in H1299 cells promoted proliferation and migration, induced G2 cell cycle arrest, and decreased apoptosis.

**Conclusions:**

This FAM-related gene signature provides a valuable tool for prognostic stratification and monitoring of TIME and immunotherapy responses in LUAD. MOGAT2 is identified as a potential anti-tumor regulator, offering new insights into its role in LUAD pathogenesis.

## 1 Introduction

Lung cancer including non-small cell lung cancer (NSCLC) and small cell lung cancer (SCLC), ranks as the leading cause of cancer-associated deaths worldwide. Lung adenocarcinoma (LUAD) is the most prevalent histological subtype, accounting for 50% - 70% of all NSCLC cases (1). Remarkable advancement in prevention and therapeutics has been made, while most patients are diagnosed at advanced stages, leading to lower 5-year overall survival. Many endeavors have been taken to develop novel therapies including molecular targeted therapy, immunotherapy, and combination therapy apart from surgery, radio/chemotherapy, and chemotherapy for NSCLC patients (2, 3). Immune checkpoint inhibitors targeting PD-1/PD-L1, and CTLA-4 have made remarkable forward in various human malignancies, while most patients are reported to obtain a short-term complete or partial remission following treatment, the majority of patients eventually raised drug resistance and succumb to tumor recurrence (4, 5). Increasing evidence witnessed that the heterogeneity of tumor microenvironment (TME) among patients, especially the variations in the infiltration of immune cells in the niche, and the interactions of tumor and host are the main determinants of responsiveness to treatment (6, 7). Emerging signatures have been proposed to predict the responsiveness to immunotherapy (8, 9), such as tumor mutation burden (TMB) (10, 11) and immune checkpoint molecule expression (12–14), while these tools are insufficient to characterize the landscape of the TME heterogeneity. This posed an unmet need to identify additional reliable biomarkers for predicting therapeutic efficacy.

Lipid metabolism abnormality is a hallmark in diverse cancers, and perturbed metabolism enabled cancer cells to acquire a rapid proliferative rate by over-activating endogenous lipid synthesis or increasing the uptake of exogenous lipids or lipoproteins (15–17). Cellular fatty acids (FAs) are involved in many biological processes including being incorporated into membrane structure, energy storage, signaling macromolecules, and oxidized into carbon dioxide for energy production. Tremendous evidence showed that FAs metabolic reprogramming in cancer has an integral role in tumorigenesis including lung cancer (18). Aberrantly activated enzymes involved in the metabolism of FAs such as ATP citrate lyase (ACLY), fatty acid synthase (FASN), and acetyl-CoA carboxylase (ACC) in normal cells accelerated cancerous transformation in lung cancer (18). Endogenous FAs metabolisms were found to be reversely correlated with EGFR expression (19) and promoted epithelial-mesenchymal-transition (EMT) regulation (20), which contributes to the invasive and metastatic capacity of lung cancer cells. Multiple molecules mediate the activity of FAs metabolism such as sterol regulatory element binding proteins (SREBPs) and FASN. High FASN expression was closely associated with decreased survival and increased cell proliferation and invasion in lung cancer (21). Inhibition of the availability and metabolism of FAs represented a promising therapeutic strategy by blocking FAs synthesis, increasing oxidative degradation, and limiting FAs release from storage (22). However, the regulatory mechanism of the FA metabolism process in LUAD has not been well interrogated. Therefore, it will provide new perspectives in understanding tumor heterogeneity and novel targets for patients with LUAD by analyzing fatty acid metabolism genes (FAMGs).

This study elucidated the prognostic utility of FAMGs in LUAD. A robust risk-stratification signature based on FAMGs was established in the TCGA cohort and predictive power was validated in six independent LUAD datasets. Signature-related features including tumor immune microenvironment (TIME) landscape, differentially expressed pathways, and immunotherapy predictive potential were delineated. We first found that low MOGAT2, the signature gene, promotes LUAD cells proliferation and migratory capability by CRISPR/Cas9 technology and *in vitro* functional assays. The study may shed insight into developing FAMGs-related targeted therapy in the treatment of LUAD by identification of risk signature.

## 2 Materials and methods

### 2.1 Lung adenocarcinoma datasets collection and pre-processing

The processed RNA-seq gene expression profile (FPKM normalized) of 535 lung adenocarcinoma (LUAD) and 59 normal samples were downloaded from the UCSC Xena database (https://xenabrowser.net/datapages) for identifying survival-related FAMGs and constructing prognostic signatures. Six independent LUAD microarrays with available clinical features including GSE30219 (n=85), GSE31210 (n=226), GSE50081 (n=127), GSE68465 (n=442), GSE72094 (n=398), and GSE11969 (n=90), were also obtained from GEO database for external validation. These publicly available datasets had ethical approval in their original studies. The details for these datasets were listed in **Supplementary Table S1**.

### 2.2 Construction and validation of FAMGs-associated prognostic signature

The FAMGs were retrieved from the MSigDB database. To investigate the prognostic roles of these FAMGs, OS-related FAMGs were identified using univariate Cox regression analysis. GO terms enrichment analysis including biological process (BP), molecular function (MF), and cellular components (CC) of these OS-related FAMGs were conducted using clusterProfiler package (23). The KEGG pathways were interrogated as well.

The OS-related FAMGs were utilized to develop a prognostic signature through multivariate Cox stepwise regression analysis. The optimal number of features for the signature was determined using both stepwise selection and the Akaike Information Criterion (AIC) (24), resulting in a formulation that included the minimum number of OS-related FAMGs along with their corresponding coefficients. FAscore for individual patient was calculated using the signature, and patients were then categorized into low- and high-risk groups based on the median risk score. Overall survival (OS) differences between these groups were assessed using the log-rank test and visualized with Kaplan-Meier curves.

A subset analysis was performed to evaluate the predictive utility of the signature across various clinical features such as age, gender, and tumor stage.

To validate the prognostic capability of the signature, it was applied to six independent LUAD datasets: GSE30219 (n=85), GSE31210 (n=226), GSE50081 (n=127), GSE68465 (n=442), GSE72094 (n=398), and GSE11969 (n=90), all of which included overall survival data.

The signature predictive capacity was assessed by the receiver operating characteristic (ROC) curve for training and validation datasets using the *survivalROC* package.

### 2.3 Predictive utility of signature for patients’ relapse

To further assess the potential of signature in predicting patient’ relapse or disease progression, the available information on disease-free survival (DFS) or first progression (FP) was collected from TCGA (n=858), GSE30219 (n=85), GSE31210 (n=226), and GSE50081 (n=124). Patients were divided into low- and high-risk groups according to the median risk score. The survival of patients in high- and low-risk groups was compared using the log-rank test.

### 2.4 Tumor immune microenvironment analysis

To characterize the signature associated TIME, the immune and stromal cell infiltration was assessed using the ESTIMATE algorithm by “estimate” package. Then, the proportions of immune cell infiltration in high- and low-risk groups were dissected using multiple cell deconvolution algorithms including CIBERSORT (25), xCell (26), TMIER (27), and EPIC (28). In addition, the expression levels of stimulatory, inhibitory checkpoint molecules, and immune senescence markers were also compared in patients within high- and low-risk groups.

### 2.5 Immunotherapy prediction

To predict the responsiveness to immune checkpoint blockade, we analyzed PD-1/PD-L1 mRNA and protein expression, Tumor mutation burden (TMB) mutant-allele tumor heterogeneity (MTH), and TCR repertoire. Gene mutation profiles of LUAD patients were downloaded from the TCGA database and TMB was calculated using “maftools” package (29). TMB was calculated as the number of somatic indels and base substitutions per million bases in the coding region of the detected genome (30). The richness and Shannon diversity indexes, which were retrieved from the Pan-Cancer Atlas study (31), were used to characterize the diversity of the TCR repertoire. The richness measures the number of unique TCRs in the sample, while the Shannon diversity index reflects the relative abundance of the different TCRs.

The Immunophenoscore (IPS) (32) quantifies tumor immunogenicity on a 0-10 scale, derived from a weighted average Z score based on key factors like MHC molecules, immunomodulators, and immune cell expression. A higher IPS suggests improved prognosis and response to immunotherapy. To forecast responsiveness to ICIs therapy, IPS data from LUAD patients treated with anti-PD-1/anti-CTLA-4 was retrieved from TCIA. IPS levels were then compared between High and Low immunity subtypes. We applied the signature to NSCLC patients receiving anti-PD-1 therapy (GSE126044) including 5 responders and 11 non-responders. The patient’s risk score was calculated and compared between responders and non-responders using the Wilcox test with p<0.05 as statistically significant.

### 2.6 Differential expression and gene set enrichment analysis

The differentially expressed genes with |Log FC| > 1 and false discovery rate (FDR) < 0.05 were identified between high- and low-risk groups by *edgeR* package (33). GSEA was conducted to identify the differentially expressed pathways between low- and high-risk patients using all the genes ranked by fold change. Hallmark and KEGG pathway gene sets were used as an enriched portal from MSigDB database (34). The adjusted *p*-value of less than 0.05 was considered statistically significant.

### 2.7 Cell culture and reagents

NCI-H1299 cells were cultured in DMEM medium supplemented with 10% fetal bovine serum, and 1% penicillin, at 37°C in a humidified atmosphere containing 5% CO2. The human NSCLC cell line NCI-H1299 was obtained from FuHeng Biology in China, RPMI-1640 medium was obtained from Servicebio (Cat: G4535, China), Fetal bovine serum was obtained from ZhengTuo Biology(Cat: 11011-8611, China), Polyethylene glycol 400(PEG400) was obtained from Solarbio (Cat: P8530, China), Dimethyl sulfoxide(DMSO) was obtained from Solarbio (Cat: D8370, China)penicillin was obtained from HaiChuang (Cat: P1400, China), Matrigel was purchased from BD (Cat: 354234, USA).

### 2.8 CRISPR/Cas9-based knockout of MOGAT2 in NCI-H1299 cells.

To create the MOGAT2-sliencing NCI-1299 cell line, CRISPR-Cas9/gRNA ribonucleoprotein complex (CRISPR-RNP) from ELEM Biotech was employed for electroporation using the Neon NxT Electroporation System (Thermo Fisher). Electroporation settings included a pulse voltage of 1300, pulse width of 10 ms, and pulse number of 3 at a cell density of 5x10^6 cells/ml. sgRNA sequence used for MOGAT2 was GCUGGUCAAGACUGCUGAGC CGACUGCCAGGACUCCAUGG, which targets exon 3 of human MOGAT2 gene with the specific cleavage site from location 75727451 to 75727494. The efficiency of knockout was confirmed through both DNA-sequencing, western blot, and RT-qPCR assays.

### 2.9 RT-qPCR

To confirm MOGAT2 knockout, total mRNA was extracted using Trizol reagent from both MOGAT2 control and KD NCI-1299 cells. Reverse transcription was performed following the protocol of the HiScript II Q RT SuperMix Kit (Vazyme, Cat: R223-01), and RT-qPCR was conducted using the Taq Pro Universal SYBR qPCR Master Mix kit (Vazyme, Cat: Q712-02). The primer sequences used were: MOGAT2 forward primer: 5’-CGGTTCTGCAGGTGTTCTTT-3’, MOGAT2 reverse primer: 5’-GGAATCCTGGGTCCGTTCA-3’, GAPDH forward primer: 5’-CCAGACAGCACTGTGTTGGCATA-3’, GAPDH reverse primer: 5’-AAAATGCAGCTTCCACCACC-3’. MOGAT2 expression was quantified by 2^–ΔΔCt^ method.

In addition, to calculate FOXM1 and MYC, which are tightly correlated with cell cycle and proliferation (35, 36), expression in MOGAT2 scramble and knockdown NCI-1299 cells, RT-qPCR was used as described above. The primers of FOXM1 and MYC were used as previously described (37, 38).

### 2.10 Western blot assay

To further assess deletion efficiency, NCI-1299 MOGAT2 knockdown and control cells were collected, lysed on liquid ice for 30 minutes, and the supernatant removed by centrifugation. Protein samples were prepared by BCA protein quantification then transferred to polyvinylidene fluoride (PVDF, Cat: IPVH00010, Millipore) membrane by SDS-POLYacrylamide gel electrophoresis (PAGE). After sealing by 5% BSA, primary antibodies against MOGAT2 (1:1000; Cat: 19514-1-AP or ab228950; ProteinTech or ab228950) and GAPDH (1:1000; cat: ab128915; Abcam) were added overnight at 4C, and corresponding peroxidase-labeled secondary antibodies (1:200; Goat anti-Rabbit IgG (H+L), Cat: G1213, Servicebio; 1:200, Goat anti-mouse IgG, Cat: G1214, Servicebio) was added. The marker ladder was used to indicate the target molecular weight. The levels of MOGAT2 protein were evaluated using chemiluminescence detection system (EMD Millipore, Billerica, USA) according to the Electrochemical luminescence (ECL) color development kit.

### 2.11 Cell counting kit-8 assay

NCI-1299-MAGAT2 CRIPSand WT control cells were seeded at 5 × 10^3 cells/well in 100 μL medium in 96-well microplates (NEST, Nest Biotech, catalog: 703001). Cell viability was assessed using Cell Counting Kit-8 (CCK8, Biosharp, Anhui, China, catalog: BS350B) following manufacturer’s instructions. 10 μL of CCK-8 reagent was added per well and incubated for 2 hours. Absorbance was measured at 450 nm using a microplate reader (Bio-Rad, Hercules, CA, USA), with wells lacking cells as blanks. Experiments were conducted in triplicate, and cell proliferation was determined by absorbance.

### 2.12 Cell apoptosis assay

MOGAT2 WT and knockdown NCI-1299 cells (5 × 10^5 per well) were seeded in 6-well plates for 24 hours following cell attachment. Subsequently, the cells were dissociated using pancreatin and rinsed with 1xPBS. Annexin V-FITC/PI apoptosis kit (Cat#: C1062M, Beyotime Biotechnology, China) was employed for apoptosis detection following manufacturer’s instructions. Finally, flow cytometry analysis (FongCyte, Challenbio, China) was conducted to assess apoptosis distribution.

### 2.13 Cell cycle assay

MOGAT2 WT and knockdown NCI-1299 cells in logarithmic growth phase were seeded at 5 × 10^5 cells/mL into 6-well plates. After 48 hours, cells were collected and washed twice with 1xPBS. Cell cycle detection kit was used (Cat: C1052, Beyotime Biotechnology, China). Briefly, cells were then digested with trypsin to ensure complete digestion, neutralized, and centrifuged at 300g for 5 minutes. The cell pellet was washed twice with pre-cold PBS. Cells were resuspended and fixed using 70% ethanol for fixation. After overnight incubation at 4°C, cells were centrifuged, washed once with 1xPBS, and treated with RNaseA at 37°C for 30 minutes. Finally, propidium iodide (PI) was added for 30 minutes at room temperature or 4°C in the dark for staining.

### 2.14 Transwell migration assay

Matrigel (1:8 dilution, Cat: 356234, Corning) was applied to Transwell inserts (Cat: TCS003024, Haote Biotech, China) and incubated at 37°C for 30 minutes to form a gel. Before use, the basement membrane was hydrated. MOGAT2 WT and knockdown NCI-1299 cells, starved in serum-free medium for 12-24 hours, were prepared at 5 × 10^5 cells/mL and added (100 μl) to Transwell chambers with 600 μl of complete medium in the lower chamber. After 48 hours, the upper chamber liquid was aspirated, and the chamber was fixed in paraformaldehyde (Cat:1950136, PiNuoFei Biotech, China) for 30 minutes, then stained with crystal violet (Cat: BL802A, Biosharp, China) for 20 minutes. After rinsing with PBS, the membrane was air-dried, mounted, and photographed at 100x, 200x, and 400x magnification (Nikon Eclipse E100) for cell counting.

### 2.15 Wound healing assay

MOGAT2 WT and knockdown NCI-1299 cells at logarithmic growth stage were seeded in 6-well plates at a density of 5 × 10^5 cells per well, with three wells per group. Once cells reached confluence, a 200 μL pipette tip was used to create a vertical scratch to avoid plate tilting. Suspended cells were then removed with 1xPBS, and plates were incubated at 37°C with 5% CO2. Microscopic images were captured at 0, 24 and 48 hours and analyzed by Image J, and the experiment was replicated three times. Cell migration rate was calculated using the formula: Migration Rate (%) = (Width of scratch at 0 hours - Width of scratch after incubation)/Width of scratch at 0 hours) × 100%. The unit of measurement is micrometers (μm).

## 3 Results

### 3.1 Identification of overall survival-related FAMGs

**Figure 1:**
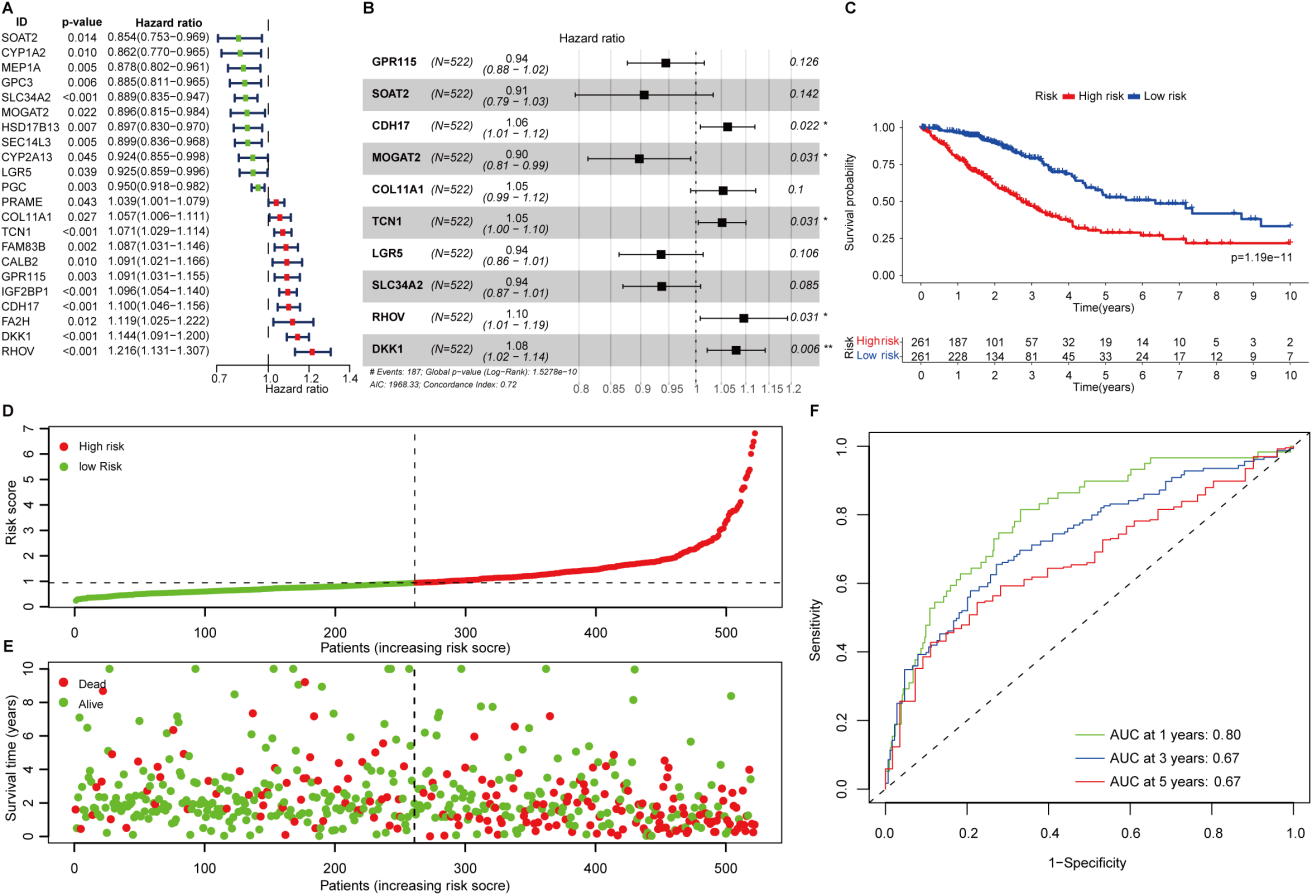
Construction of fatty acid metabolism associated prognostic signature. **(A)** Forest plot showing the overall survival-related fatty acid metabolism genes in LUAD. **(B)** The signature formula. **(C)** The Kaplan-Meier curve shows the survival difference of low- and high-risk patients. **(D)** Distribution of risk scores among LUAD patients. **(E)** The number of deaths varies with increasing risk scores. **(F)** 1-, 3-, and 5-year of AUCs value of the signature.

To investigate the biological process of the FAMGs, functional enrichment analysis showed that these FAMGs were enriched in the cellular response to stimulus and metabolic-related biological processes such as steroid metabolism, bile acid, and epoxygenase P450 pathway (**Supplementary Figure S1A**). KEGG analysis confirmed that cytochrome P450 metabolism, Wnt, steroid, Phenylalanine, and bile acid synthesis ranked as the top pathways (**Supplementary Figure S1B**). Then, we explored the interplay of these FAMGs through PPI network analysis, and two modules (named CYP3A4, and KRT19) were identified by the number of nodes (**Supplementary Figure S1C**). In module CY3A4, these membrane-associated CYPs are the major enzymes involved in drug metabolism. Genes in module KRT19 are the regulators in Wnt signaling which control cell proliferation and growth. This further promoted us to assess their clinical relevance in LUAD. A total of 22 FAMGs were found to be correlated with patients’ outcomes by univariate Cox regression analysis in the TCGA LUAD dataset (**Figure 1A**). Among these OS-related FAMGs, decreased survival was observed in patients with high expression of 11 OS-related FAMGs such as TCN1, RHOV, DKK1, and GPR115, while elevated expression of the remaining FAMGs was considered as protective factors.

### 3.2 Construction and validation of the prognostic signature based on FAMGs

The 22 FAMGs with prognostic significance were delivered for further analysis. Stepwise multivariate Cox proportional hazard regression analysis was employed to determine the minimum set of features that generated the most powerful prediction for patients’ prognoses. This identified 10 genes of significance (GPR115, SOAT2, CDH17, MOGAT2, COL11A1, TCN1, LGR5, SLC34A2, RHOV, and DKK1) which comprise the optimal prognostic signature (**Figure 1B**). The formulation was listed as follows:

FAScore = [Expression level of *GPR115* *(-0.0573)] + [Expression level of SOAT2*(-0.0987)] + [Expression level of *CDH17* *(0.0608)] + [Expression level of *MOGAT2* *(-0.1079)] + [Expression level of *SLC34A2* *(-0.0651)] + [Expression level of *COL11A1* *(0.0522)] + [Expression level of *TCN1* *(0.0502)] + [Expression level of LGR5*(-0.0663)] + [Expression level of *RHOV* *(0.0917)] + [Expression level of *DKK1* *(0.0771)]

The FAScore for an individual patient was calculated, and patients were divided into low- and high-risk groups using median FAScore as the cutoff value. We generated a Kaplan-Meier curve and decreased survival was observed in patients within the high-risk group as compared to those patients in the low-risk group (**Figure 1C**, p=1.19e-11). The number of deaths was increasing along with elevated FAScore (**Figures 1D, E**). We noted that six model genes (GPR115, CDH17, MOGAT2, COL11A1, TCN1, and RHOV) were significantly up-regulated in LUAD patients as compared to adjacent normal tissues (**Supplementary Figure S1D**), while two genes (SLC34A2 and LGR5) were markedly down-regulated in patients (**Supplementary Figure S1D**). Additionally, six model genes were up-regulated in high-risk patients (**Supplementary Figure S1E**), while the expression of the remaining four genes (SLC34A2, LGR5, MOGAT2, and SOAT2) was observed to be increased in low-risk patients (**Supplementary Figure S1E**). The area under curve (AUC) values of the ROC curve for 1-, 3-, and 5-year were 0.80, 067, and 0.67 (**Figure 1F**), indicating that the prognostic signature has a robust capacity for monitoring prognosis.

**Figure 2:**
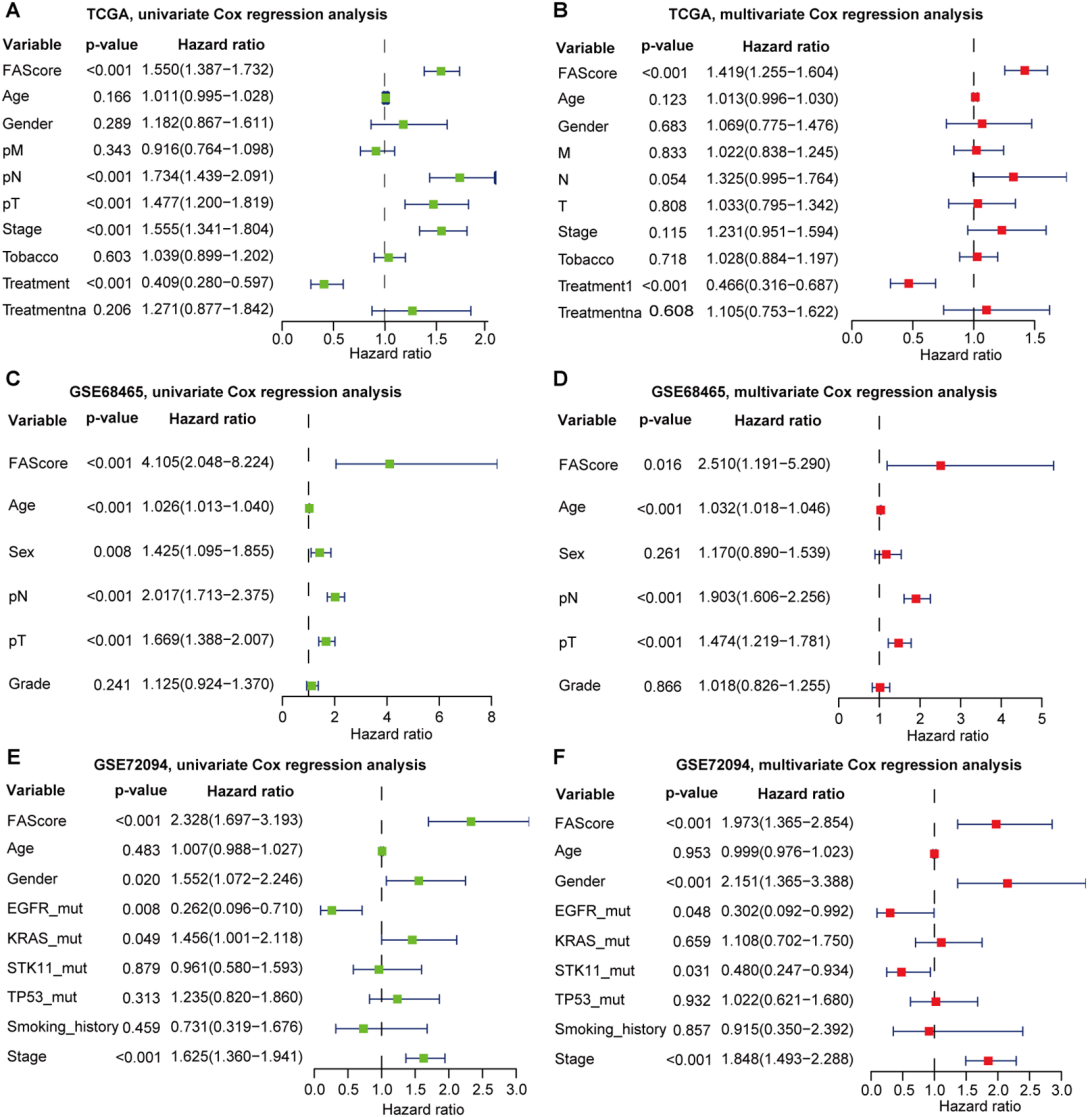
Univariate and multivariate Cox regression analyses of the signature by incorporating clinical features. **(A)** Univariate Cox regression analysis of the signature in the TCGA LUAD cohort. **(B)** Multivariate Cox regression analysis of the signature in the TCGA LUAD cohort. **(C)** Univariate Cox regression analysis of the signature in the GSE68465 set. **(D)** Multivariate Cox regression analysis of the signature in the GSE68465. **(E)** Univariate Cox regression analysis of the signature in the GSE72094 set. **(F)** Multivariate Cox regression analysis of the signature in the GSE72094 set.

Clinicopathological features such as clinical stages and age were correlated with disease progression and patient’s prognosis. To further test its predictive independence, univariate Cox regression analysis was performed in the TCGA LUAD dataset, and we found that FAScore, involved lymph nodes, tumor size, and clinical stages correlated with decreased survival, while patients receiving treatment have favorable survival (**Figure 2A**). Multivariate Cox regression analysis showed that the prognostic signature could serve as an independent predictor after adjusting for other clinical parameters such as treatment (**Figure 2B**). Similar observations were found in the GSE68465 LUAD dataset (**Figures 2C, D**). In the GSE72094 dataset, the FAScore could serve as an independent indicator for OS prediction (**Figures 2E, F**). In addition, patients with oncogenic driver mutations including EGFR and KRAS were associated with clinical survival, which was consistent with previous reports.

### 3.3 Validation of the prognostic signature in independent datasets

**Figure 3:**
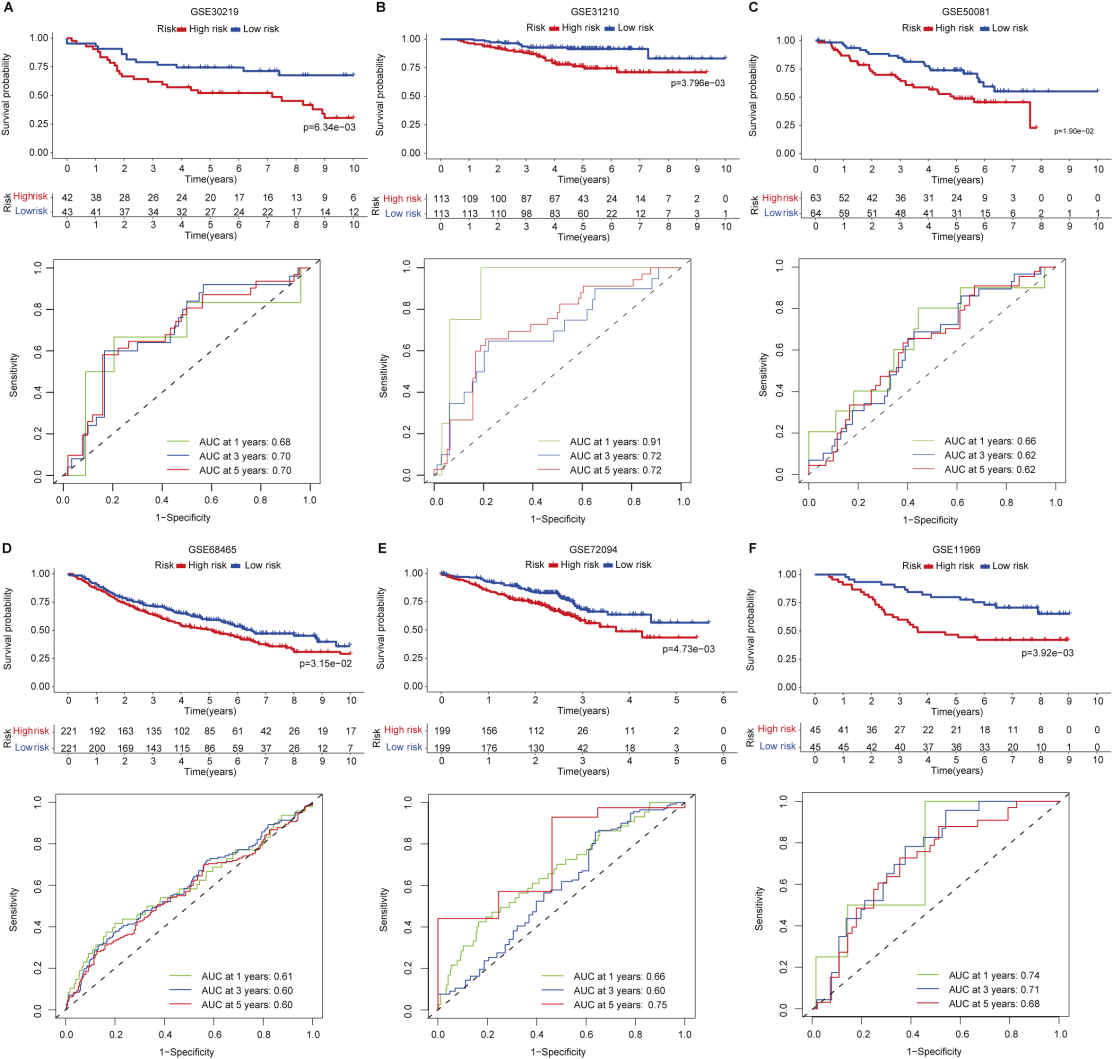
Signature validation in six independent LUAD cohorts. **(A)** GSE30219. **(B)** GSE31210. **(C)** GSE50081. **(D)** GSE68465. **(E)** GSE72094. **(F)** GSE11969.

To verify the reproducibility of the prognostic signature in LUAD, we applied this signature formula in six independent publicly available cohorts (GSE30219 (n=85), GSE31210 (n=226), GSE50081 (n=127), GSE68465 (n=442), GSE72094 (n=398), and GSE11969 (n=90)) totaling 1,368 LUAD patients to assess the predictive capability of the proposed signature for patients’ prognosis. Patients were also stratified into low- and high-risk groups, and we found that patients in the low-risk group in these six cohorts have prolonged OS than those in the high-risk group (**Figures 3A–F**). The predictive performance of the signature in each validation set was also calculated and showed that the AUCs of 1-, 3-, and 5-year all exceeded 0.6. Specifically, the AUC of 1-year in the GSE31210 validation set was 0.91 (**Figure 3B**). These data convinced that the signature has robust risk stratification in the microarray-based platform for LUAD patients (**Figure 3A–F**).

### 3.4 Differentially expressed and functional enrichment analysis

The differentially expressed genes (DEGs) were identified using the *edgeR* package. To look at the pathways involved in the difference of the malignant characteristics between low- and high-risk groups, Gene Set Enrichment Analysis (GSEA) was performed in Hallmark and KEGG pathway gene sets. Epithelial-mesenchymal-transition (EMT), DNA repair, oxidative phosphorylation, G2M checkpoint, and hypoxia were found to be enriched in high-risk patients (**Supplementary Figures S2A–D**, **Supplementary Table S2**), suggesting dysregulated metabolism and cell cycle, and the malignant transition was active in patients within the high-risk group. These pathways were also observed in the KEGG gene set enrichment analysis (**Supplementary Figures S2E–H**). Increasing reports indicated that these pathways were correlated with resistance to ICB therapy (39).

### 3.5 Predictive potential of the signature for patients’ relapse

**Figure 4:**
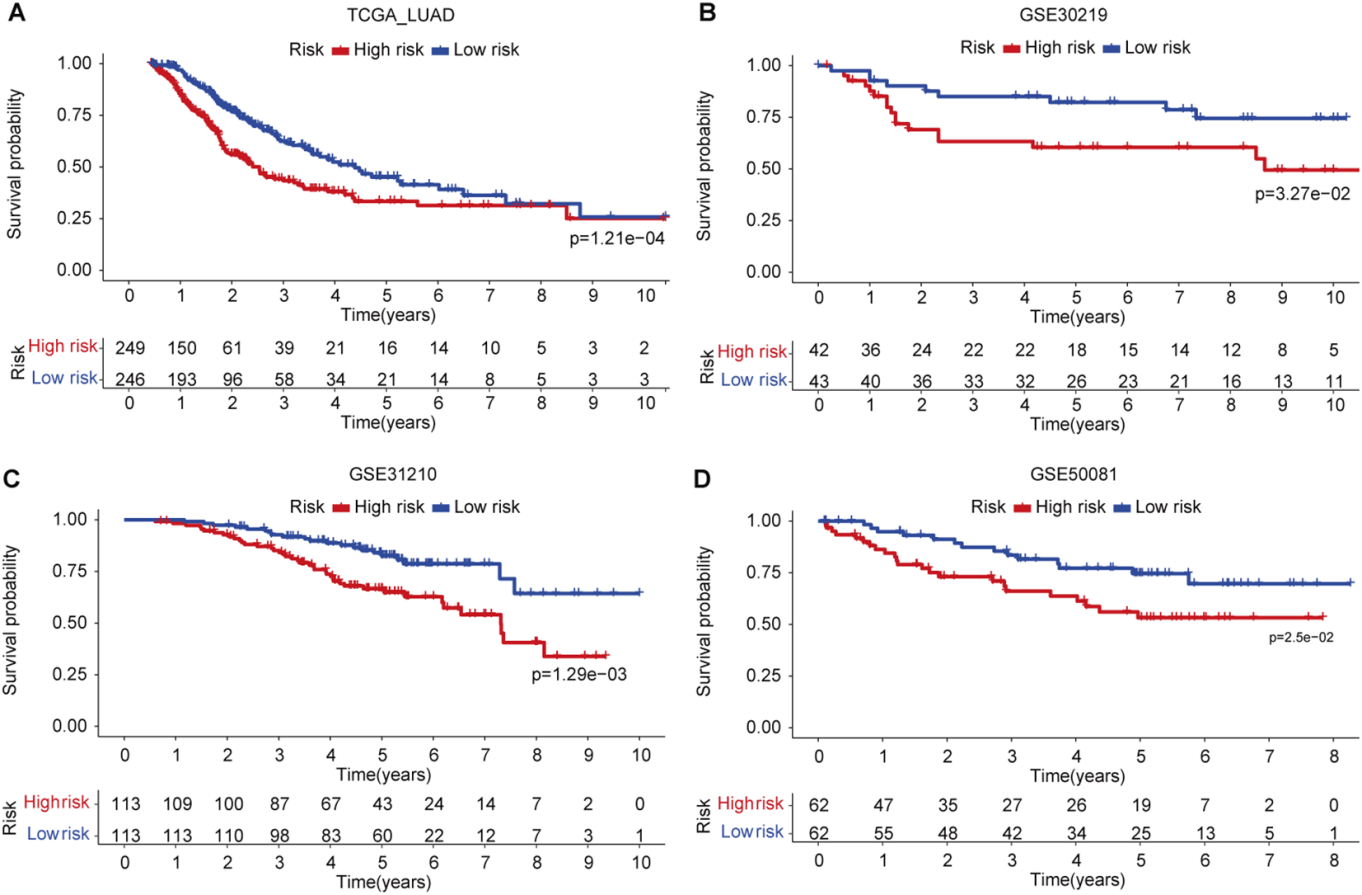
The predictive capacity of the signature for relapse or disease progression. **(A)** Kaplan-Meier curve of patients’ relapse risk in low- and high-risk groups in TCGA LUAD cohort. **(B)** Kaplan-Meier curve of patients’ relapse risk in low- and high-risk groups in GSE30219 set. **(C)** Kaplan-Meier curve of patients’ relapse risk in low- and high-risk groups in GSE31210 set. **(D)** Kaplan-Meier curve of patients’ relapse risk in low- and high-risk groups in GSE50081 set.

Relapse has been one of the main challenges for patients receiving various types of treatment such as chemotherapy or radiotherapy. We used four LUAD data sets (TCGA, GSE30219, GSE31210, and GSE50081) with available clinical information on patients’ relapse or progression to investigate the predictive potential of the signature. We found that patients in the low-risk group have significantly favorable progression-free survival as compared to those patients in the high-risk group (**Figure 4A**). A similar result was also observed in the GSE30219 set (**Figure 4B**). As for disease relapse, patients with low FAScores in the GSE31210 set showed better survival (**Figure 4C**), suggesting the signature may be an indicator for monitoring patients’ relapse or disease progression, which was verified in an independent LUAD set (GSE50081) as well (**Figure 4D**).

### 3.6 Tumor immune microenvironment analysis

**Figure 5:**
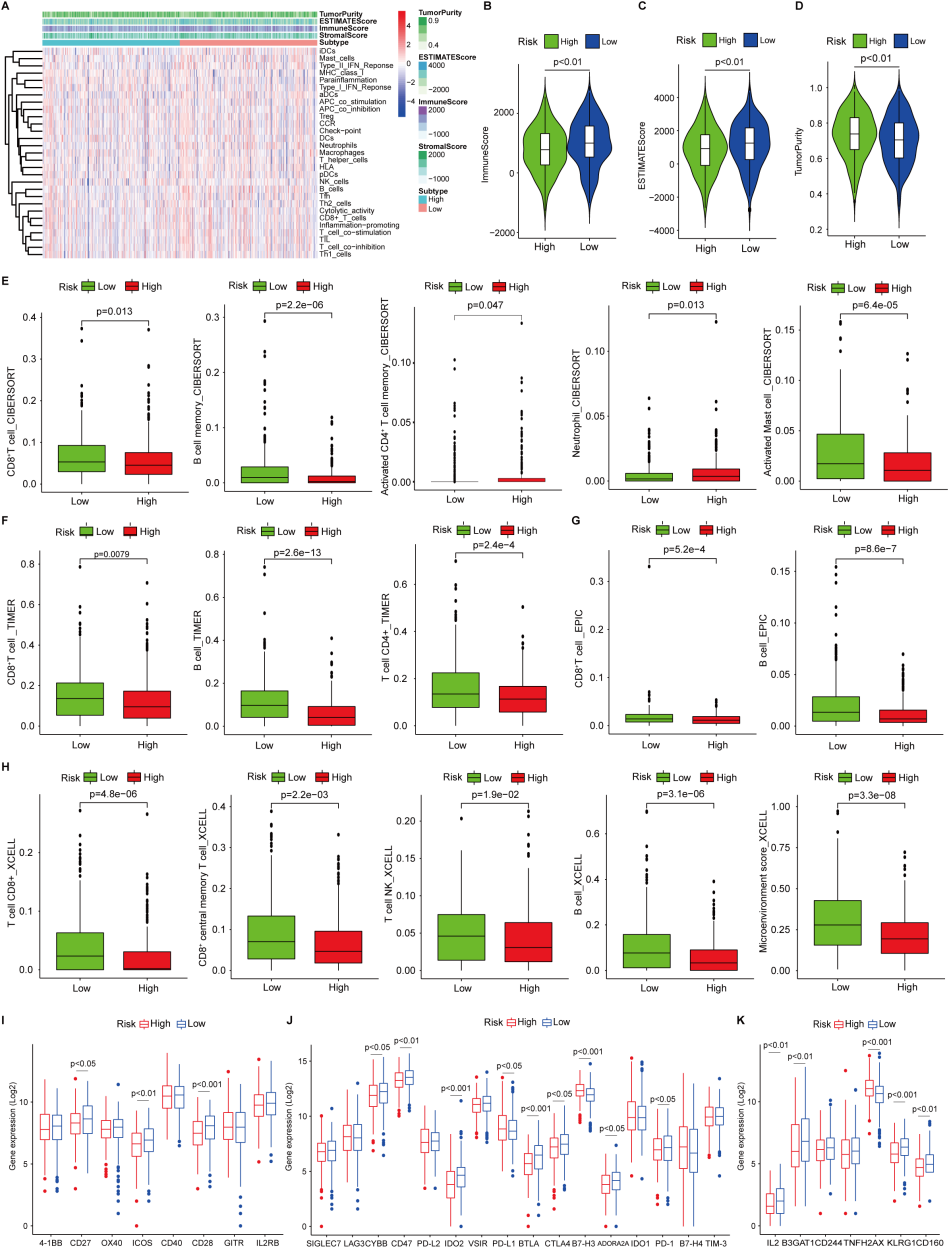
Tumor immune microenvironment landscape analysis. **(A)** single sample gene set enrichment analysis of 29 immune signatures in low- and high-risk patients. **(B, C)** Immune scores and ESTIMATEscores in low- and high-risk patients analyzed by ESTIMATE algorithm. **(D)** The tumor purity in low- and high-risk patients. **(E)** Infiltrated CD8^+^ T cells, B cells, activated CD4^+^ T cells, neutrophils, and activated mast cells in low- and high-risk patients analyzed by CIBERSORT. **(G)** Infiltrated CD8^+^ T cells and B cells in low- and high-risk patients analyzed by EPIC **(H)** Infiltrated CD8^+^/central memory CD8^+^ T cells, NK cells, B cells, and microenvironment scores in low- and high-risk patients analyzed by *xCell*. **(I, J)** Co-stimulatory and co-inhibitory receptor molecule expression in low- and high-risk patients. **(K)** The markers of cellular senescence expression in low- and high-risk patients.

Recognition of the dual role of TIME in anti-tumor immunity has led to remarkable leaps forward in tumor immunotherapy) (40). To delineate the TIME landscape in both risk groups, each patient was scored based on profiling of 29 immune signatures using single sample gene set enrichment analysis (ssGSEA) and found that patients in the low-risk group showed enhanced immune activities (**Figure 5A**). Further ESTIMATE analysis showing higher immune scores and ESTIMATE scores, and decreased tumor purity (**Figures 5B–D**) in patients within the low-risk group in contrast to the high-risk group demonstrated the notion (**Figures 5B, C**). Next, we interrogated the infiltrated immune cell subsets by multiple deconvolution algorithms. CIBERSORT analysis showed that CD8^+^ T cells and B cells increased in patients in the low-risk group, whereas activated CD4^+^ T cells, neutrophils, and mast cells were elevated (**Figure 5E**), which was confirmed by TIMER (**Figure 5F**) and EPIC (**Figure 5G**) infiltration analysis. In addition, *xCell* deconvolution also convinced that CD8^+^ and CD8^+^ central memory T cells, and microenvironment scores were higher than those of patients in the high-risk group (**Figure 5H**). Several co-stimulatory molecules were up-regulated in low-risk patients such as CD27/28, and ICOS (**Figure 5I**), while the co-inhibitory molecules were shown differential expressed in both groups, CD47, IDO2, CTLA4, and PD-1, were increased expression in low-risk patients, but PD-1/L1 and B7-H3 were decreased (**Figure 5J**), indicating that patients in low-risk groups might benefit from the immunotherapy targeting PD-1/CTLA4. We noted that IL2, CD160, and KLAG1, the positive regulators of immune cell proliferation and activation expressed highly in patients in the low-risk group, and H2AX, a key immune cell senescence marker, expressed higher in high-risk patients, this might be a sign of immune cell exhaustion in this group (**Figure 5K**).

### 3.7 The signature correlates with anti-tumor immunity and therapy response

**Figure 6:**
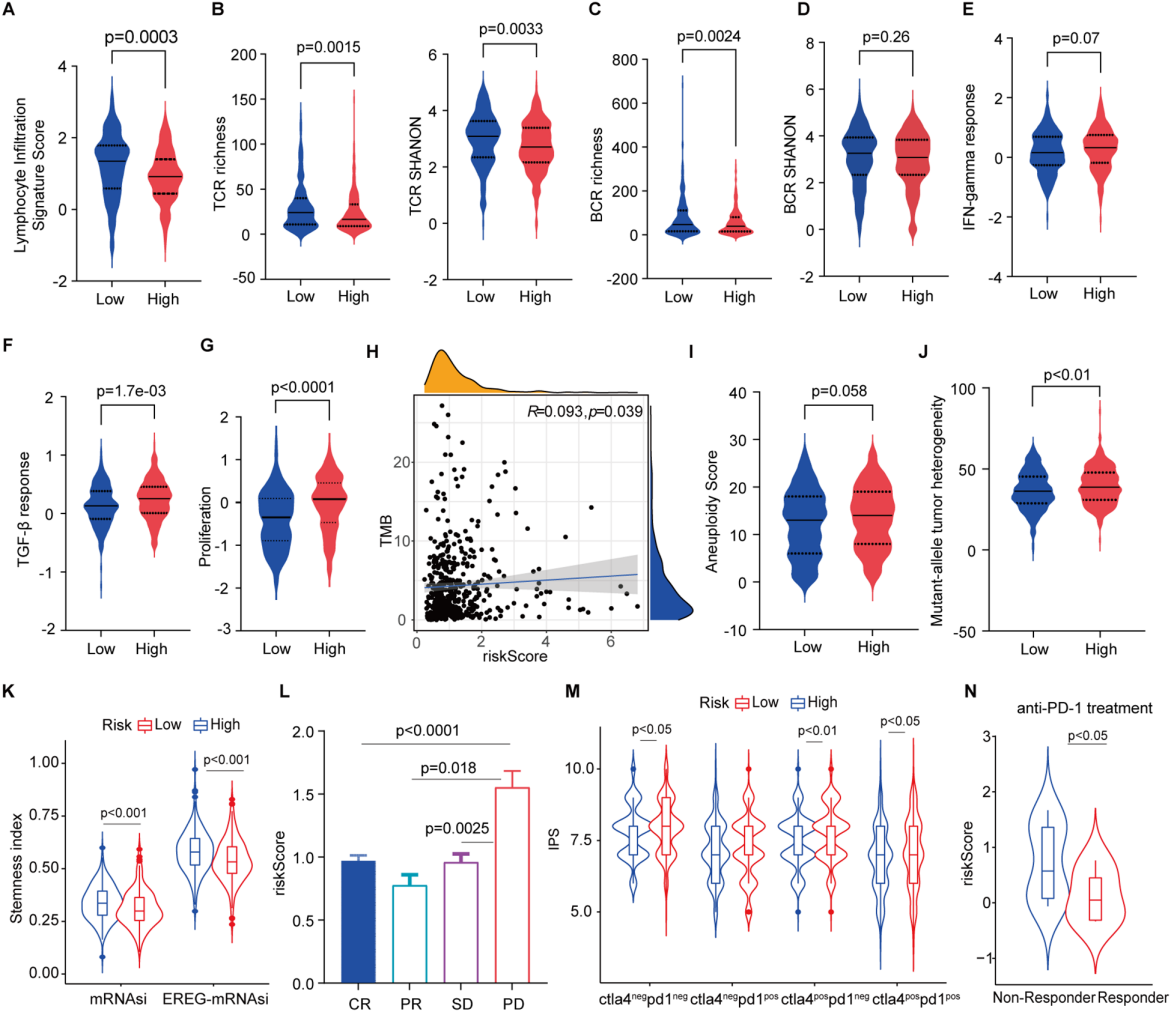
Association of the signature with anti-tumor immunity and immunotherapy. **(A)** Lymphocyte infiltration signature score in low- and high-risk patients. **(B)** T cell receptor richness and diversity in low- and high-risk patients. **(C, D)** B cell receptor richness and diversity in low- and high-risk patients. **(E)** IFN-gamma and TGF-β response scores in low- and high-risk patients. **(G)** Proliferation index in low- and high-risk patients. **(H)** Correlation of tumor mutation burden with risk scores. **(I, J)** Aneuploidy score and mutant-allele tumor heterogeneity in low- and high-risk patients. **(K)** Stemness index in high- and low-risk patients. **(L)** risk score distribution in responders and progressive disease. **(M)** IPS score in low- and high-risk patients. **(N)** Risk scores non-responders and responders in patients receiving anti-PD-1 treatment.

Given the difference of elevated tumor-infiltrating lymphocytes (TILs) (**Figures 5E–H**, **6A**) such as cytotoxic T cells and NK cells in high- and low-risk groups and their critical roles in predicting the efficacy and treatment response. We investigated the association between the signature and widely used immunotherapy markers PD-L1 expression (**Figure 5J**) and tumor mutation burden (TMB) in the LUAD cohort. FAScore was positively correlated with the TMB of patients (**Figure 6J**). Accounting reports have revealed that the repertoire of T cell receptors (TCR), which recognize antigens presented by the major histocompatibility complexes (MHC), could serve as a predictive indicator of responsiveness to immunotherapy (41, 42). We conducted the repertoire analysis of TCR and found that patients in the low-risk group exhibited higher TCR richness and diversity (**Figure 6B**). B cell receptor richness in low-risk patients was also increased (**Figure 6C**), while the BCR diversity was comparable (**Figure 6D**).

IFN-γ is a pleiotropic cytokine with antitumor or pro-tumorigenic roles (43), and TGF-β is an important cancer-promoting cytokine that contributes to the suppression of anti-tumor immunity (44). We subsequently scored the IFN‐γ and TGF-β responses and found that both cytokine responses were enhanced in high-risk patients (**Figures 6E, F**). The proliferation index was accordingly markedly higher in patients with high-risk scores (**Figure 6G**). In addition, we found that FAScore was significantly correlated with TMB (**Figure 6H**). A lower aneuploidy score has been observed in patients with complete or partial responses to immune checkpoint blockade (45), and the same decreased trend was found in low-risk patients as compared to those in high-risk patients (**Figure 6I**). Mutant-allele tumor heterogeneity (MATH), a hallmark of cancer that is a promising biomarker for clinical outcomes and patients’ response to therapy (46), was decreased in low-risk patients in contrast to those high-risk patients (**Figure 6J**). Furthermore, the number of cancer stem cells (CSCs) was estimated using an mRNA expression-based stemness index and found that CSCs was decreased in low-risk patients (**Figure 6K**).

Assessment of responsiveness to therapy including immunotherapy is a critical challenge before treatment. These identified features supported that the signature implies predicting responsiveness to therapies. We found that patients with progressive disease have higher FAScores than those patients with responders after receiving chemo/radiotherapy in the TCGA dataset (**Figure 6L**). IPS analysis indicated that low-risk patients might benefit from the anti-PD-1/CTLA-4-based immunotherapies (**Figure 6M**). Next, to verify the hypothesis, we applied the signature to the dataset from patients receiving anti-PD-1 treatment and found that responders have significantly lower risk scores as compared to patients in the high-risk group (**Figure 6N**).

### 3.8 MOGAT2 deficiency promotes proliferation of LUAD cells

The roles of several signature genes, including GPR115 (47), TCN1 (48), COL11A1 (49), RHOV (50), DKK1 (51), SLC34A2 (52), LGR5 (53), SOAT2 (54), and CDH17 (55), in the tumorigenesis of NSCLC have been well established, with some emerging as potential therapeutic targets. However, the role of MOGAT2 in NSCLC progression remains unexplored, prompting our investigation. We found that MOGAT2 expression was higher in low-risk patients as compared to that in the high-risk patients (**Supplementary Figure S1E**). We hypothesized that silencing MOGAT2 would enhance NSCLC growth, considering its association with improved survival when expressed at high levels.

**Figure 7:**
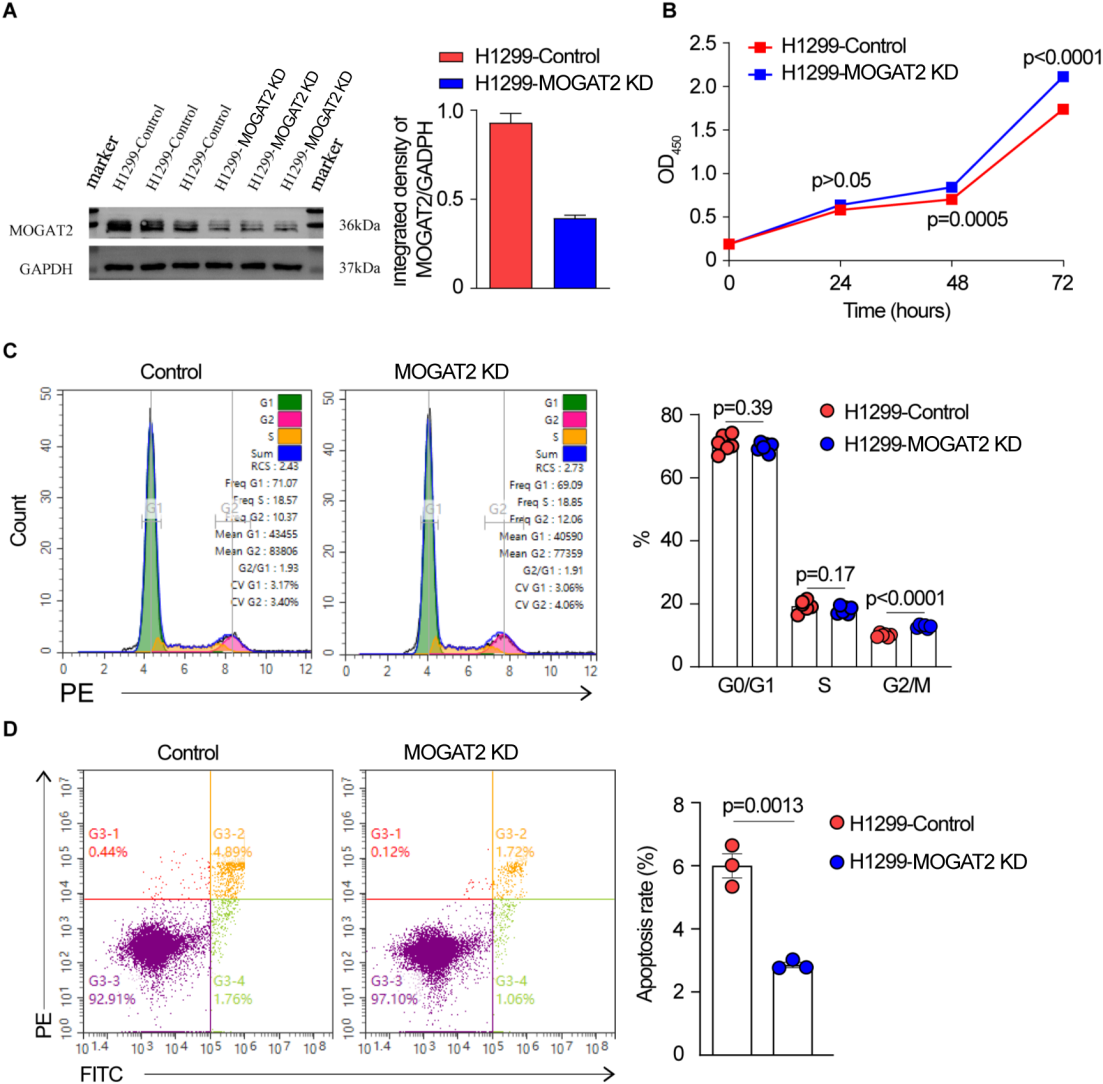
The effect of MOGAT2 on the proliferation of H1299 cells. **(A)** Western blot verify MOGAT2 knockdown in CRISPR/Cas9 edited MOGAT2 knockdown and control H1299 cells. **(B)** The cell proliferation of NCI-1299 MOGAT2 knockdown and H1299 control cells using CCK8 assay. **(C)** Cell cycle analysis of NCI-1299 MOGAT2 knockdown and H1299 control cells. **(D)** Cell apoptosis of NCI-1299 MOGAT2 knockdown and H1299 control cells.

Using CRISPR/Cas9 technology, we deleted MOGAT2 in H1299 cells and confirmed its deficiency through Western blot, DNA sequencing, and RT-qPCR (**Figure 7A**; **Supplementary Figures S3A, B**, **S4A**). MOGAT2 expression was significantly knocked down (KD). We observed that the levels of the three different isoforms were reduced to varying degrees, which was confirmed by repeating the Western blot analysis with a different anti-MOGAT2 antibody in CRISPR/Cas9 edited H1299 cells (**Supplementary Figure S4B**). Further investigation into how this sgRNA affects these isoforms will be needed in future studies. The proliferative ability of these cells was assessed using the CCK8 assay, which revealed a significant increase in cell number following MOGAT2 (**Figure 7B**). Cell cycle analysis further indicated that MOGAT2 KD promoted G2 phase arrest rather than G1 and S stages (**Figure 7C**). Correlation analysis of MOGAT2 expression with the cell proliferation markers FOXM1 and MYC, using the TCGA dataset, indicated a negative correlation between MOGAT2 and both FOXM1 and MYC expression (**Supplementary Figure S5A**). Moreover, in MOGAT2-KD H1299 cells, the expression levels of FOXM1 and MYC were increased compared to control H1299 cells using RT-qPCR (**Supplementary Figure S5B**). This finding is consistent with previous research, which reported that MOGAT2 deficiency promotes colorectal carcinoma growth by activating the NF-κB pathway (56). Additionally, MOGAT2 deficiency resulted in a reduction in cell apoptosis compared to the control group (**Figure 7D**). These findings suggest that high MOGAT2 may play a role in inhibiting LUAD proliferation.

### 3.9 MOGAT2 inhibits migratory capabilities of LUAD cells

**Figure 8:**
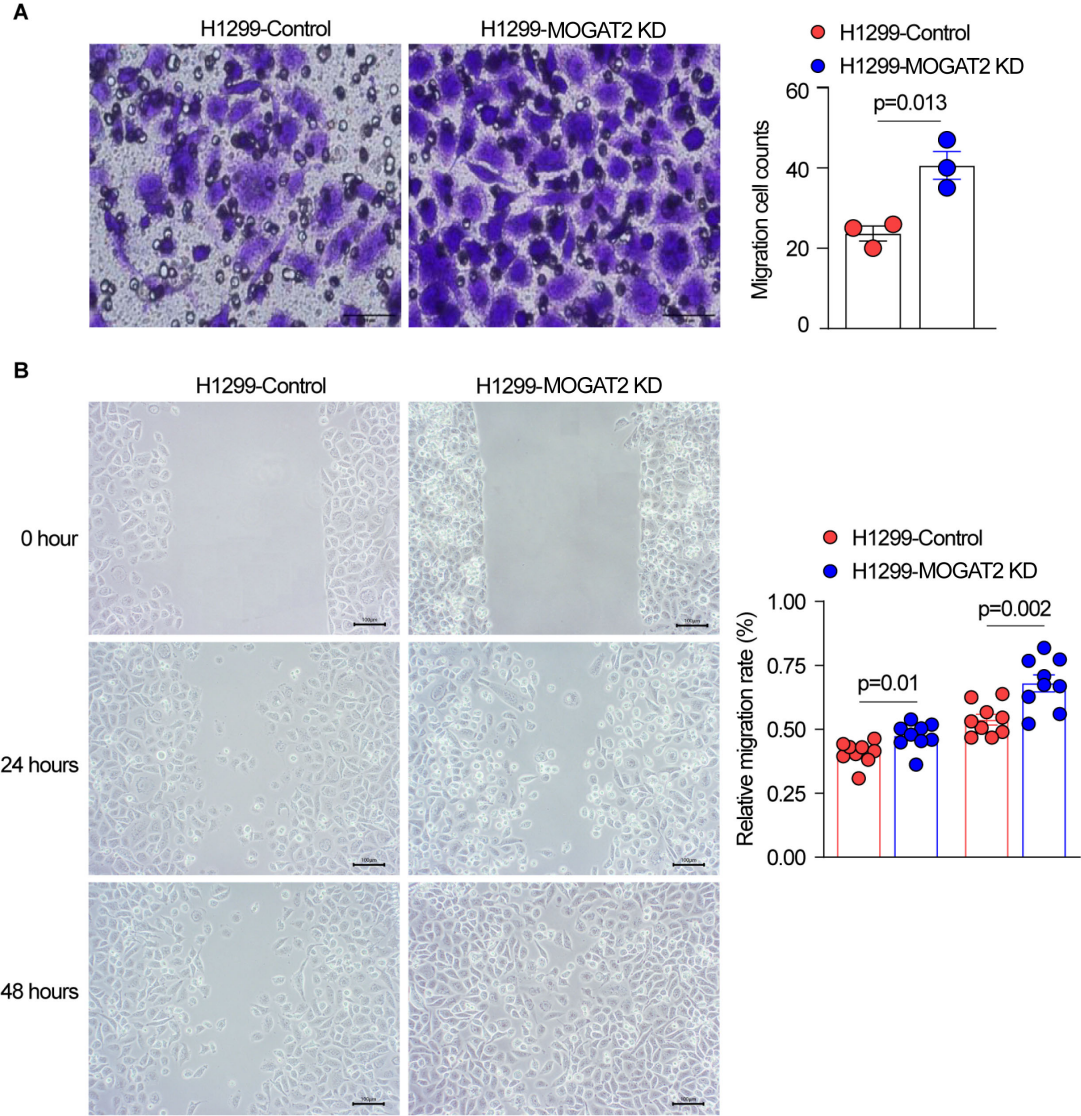
The effect of MOGAT2 on the migratory capability of H1299 cells. **(A)** Migratory capability of NCI-1299 MOGAT2 knockdown and H1299 control cells tested using transwell assay. **(B)** Migration rate of NCI-1299 MOGAT2 knockdown and H1299 control cells tested using Wound healing assay.

To determine the effect of MOGAT2 on the migratory capabilities of LUAD cells, transwell migration and wound healing assays were conducted. Transwell migration assays demonstrated a significant increase in the migration of H1299 cells with MOGAT2 knockdown compared to the control group (**Figure 8A**). Similarly, wound healing assays showed that the migration rate of H1299 cells with MOGAT2 KD was higher than that of the control group (**Figure 8B**). These data suggest that MOGAT2 KD promotes the migration of H1299 cells.

### 3.10 Potential impact of MOGAT2 in the tumor microenvironment

To explore the potential impact of MOGAT2 in the tumor microenvironment, we conducted a Spearman correlation analysis, revealing a positive correlation between MOGAT2 expression and both the immune score and the overall TME score (**Supplementary Figure S6A**). This finding suggests that MOGAT2 may be involved in the immune response.

Next, we used CIBERSORT and TIMER to analyze the fractions of infiltrating immune cell subsets, followed by a correlation analysis between MOGAT2 expression and these subsets. We observed a positive correlation between MOGAT2 expression and the presence of B cells, CD4^+^ T cells, myeloid dendritic cells, and neutrophils (**Supplementary Figures S6B, C**). These results suggest that MOGAT2 may play a significant role in shaping the TME during disease progression. However, further validation using *in vivo* mouse models is necessary to confirm these findings.

## 4 Discussion

Lung adenocarcinoma is one of the most frequent histological subtypes of NSCLC. Great advancements in high-throughput genomics studies have been made in recent years which accelerate the understanding of tumor heterogeneity (57). Although patients with LUAD benefited from the emerging treatments including molecular targeted therapy, anti-PD-1/CTLA4 immunotherapy, and chimeric antigen receptor (CAR) T cell therapy, the remission duration is limited. Increasing evidence indicated that the tumor microenvironment context matters in anti-cancer immunity, while few biomarkers that predict immune therapy responses and prognoses could delineate the TME in LUAD. Dysregulated fatty acid metabolism in cancer can serve as the essential accomplice in cancer progression and metastasis by reprogramming the TME (58). Fatty acid (FA) *de novo* synthesis is abnormally activated in cancer cells to meet the energy demands of rapid proliferation (59). In addition to FA *de novo* synthesis, the aberrant activation of FA desaturation and prolongation are common features of cancer. As a metabolic enzyme regulating FAs. Stearoyl-CoA desaturase (SCD) converts FA into MUFA, converts FAs into monounsaturated FAs (MUFA), enhancing lipid utilization in tumors and regulating the SFA/MUFA ratio in the TME (60). SCD1 inhibitors boost CD8^+^ T cell production and inhibit colon and non-small cell lung cancer growth, working synergistically with anti-PD-1 antibodies (61). Fatty acid desaturase (FADS) enzymes convert FAs into polyunsaturated FAs (PUFA). Increased FADS2 expression is linked to hepatocellular carcinoma (HCC) and non-small cell lung cancer. Inhibiting FADS2 and SCD suppresses tumor growth (62). Thus, targeting fatty acid metabolism has become a promising therapeutic strategy in fighting cancer (63), whereas the roles of fatty acid metabolism-associated genes in LUAD have not been fully investigated. In this study, we systematically explored the associations between the gene expressions of FAMGs and clinical outcomes in LUAD patients. We identified a robust FAMGs-related risk signature that is tightly correlated with the OS and DFS of patients. This signature was validated in six publicly independent available LUAD cohorts. The utility of the signature was further confirmed as an independent indicator for patients’ prognosis by adjusting for clinical features. The relevant mechanisms of the signature in predicting tumor microenvironment landscape, anti-immunity regarding antigen-specific tumor killing, and responsiveness to immunotherapy were also analyzed. Additionally, the role of signature gene MOGAT2 in LUAD was firstly investigated by experimental assays and indicated that it inhibits LUAD growth. Therefore, the proposed signature may shed light on monitoring outcomes and understanding personal immunotherapy for patients with LUAD.

The biological role and therapeutic potential of fatty acid metabolism have attracted interest in cancer, and rare comprehensive evaluations of their clinical relevance in LUAD were reported. We found that they have diverse effects on prognosis by Kaplan-Memier curve analysis, indicating that differential expression of these FAMGs has implications for cancer progression. Overexpression of Type XI collagen (COL11A1) promoted cell proliferation, migration, and drug resistance in NSCLC or recurrent NSCLC by *in vitro* and *in vivo* functional assays (49, 64). Patients with high COL11A1 correlated with decreased survival was consistent with the notion that it is a prognostic biomarker for various cancers including NSCLC (65). Similar functions were observed for IGF2BP1 (66), FAM83B (67), GPR115 (47), TCN1 (48), and PRAME (68) in NSCLC. It might be a novel druggable target for COL11A1-high cancers. RHOV has been identified as one of the most up-regulated Rho GTPase members in lung adenocarcinoma and was associated with unfavorable survival by bulk and single RNA sequencing (69). RHOV silencing inhibited proliferation and migration, as well as improved the sensitivity of EGFR-TKI and increased cancer cell apoptosis in gefitinib-resistant PC9 cells (50). In addition, high Dickkopf-1 (DKK1), an inhibitor of the Wnt/β-catenin signaling pathway, in NSCLC was linked with the proliferative and invasive capacity, and it could be a potential therapeutic target (51). CDH17 was considered as a tissue-specific diagnostic marker for adenocarcinomas (55). Thus, further investigation of these tumors promoting FAMGs in mediating metabolism in the TME might represent promising therapeutics for patients with LUAD. This prompted us to systematically profile these genes in LUAD by mathematical modeling, which has been widely employed to monitor outcomes or predict treatment response in recent years. Many tools have been in the preclinical phase or approved by FDA after multi-centers sets validation (70), such as Guardant360^®^ CDx, a qualitative next-generation sequencing-based diagnostic device that uses targeted high throughput hybridization-based capture technology for detecting mutations of 55 genes to identify non-small cell lung cancer patients who may benefit from treatment with the targeted therapies (71). This study confirmed that FAMGs are important predictors of survival among LUAD patients. We constructed a robust signature with great prognostic value for OS prediction utilizing TCGA RNA-sequencing data as the training set. The reliability of the signature was then verified via multiple diverse microarray platforms, suggesting that it has powerful risk discrimination in pooled populations and strong translational potential. The signature still showed superior prognostic utility when integrated clinical characteristics such as tumor stage, grade, and oncogenic drivers’ mutations. Its predictive performance was also confirmed by ROC curve analysis exhibiting moderate to high AUC values.

Secreted FAs accumulation in TME promoted the infiltrated immune cell function and phenotype. Abnormal fatty acid metabolism including fatty acid oxidation and lipid synthesis would nourish cancer cell survival, increase resistance to chemotherapeutic/radiation treatments, and weaken cellular stresses (18, 72). Tumor immune microenvironment (TIME) analysis indicated elevated immune scores in low-risk patients. Further immune cell subset deconvolution by different algorithms confirmed that higher infiltrated total T cells, central memory CD8^+^/CD8^+^ T cells, B cells, and NK cells in low-risk patients in opposite to that of high-risk patients. These tumor-infiltrating lymphocytes (TILs) are the main killers in anti-tumor immunity. Amounting reports noted that these TILs usually are less located in tumor sites and growing exhaustion (73, 74). Many co-stimulatory and inhibitory molecules were found increased expression in low-risk patients, as well as T cell activation markers such as KLRG1, which might imply a hyporesponsive state of T cells in tumor-killing activities that decrease tumor burden to improve survival. In addition, patients with risk scores may benefit from immune checkpoint blockade (ICB). Meanwhile, markers of T cell senescence including CD57 and H2AX were increased in high-risk patients, indicating dysfunctional protective immunity (75). Low precursor frequency and T cell receptor (TCR) affinity have been demonstrated in the most tumor-specific T cells, and antigen presentation was also impaired in TME, which result in weak priming and boosting of T cells (76). TCR, a unique molecule on the T cell surface, can recognize antigens presented by MHC, and it has been considered as a potent indicator to predict immunotherapy response (77, 78). The effectiveness of ICB therapy was tightly correlated with the abundance and functionality of infiltrated T cells in the tumoral niche. The richness and diversity of TCR repertoire were assessed and we found that low-risk patients showed higher TCR richness and diversity, which indicated enhanced T cell functionality in recognizing antigens and killing tumor cells. Additionally, biomarkers of ICB response PD-L1 were increased in high-risk patients, while low-risk patients harbored significantly lower TMB, suggesting decreased immunogenicity in low-risk tumors. IPS analysis confirmed that low-risk patients might be sensitive to ICB. Therefore, we validated the predictive value of the signature using an NSLCLC cohort that received anti-PD-1 treatment and found that responders have lower risk scores in contrast to those of non-responders. This might explain why the functionality of TILs is more important than immunogenicity. Overall, low-risk patients were prone to benefit from ICB. Further validation in more cohorts will convince the signature is a reliable marker for immunotherapy response.

To seek differentially expressed pathways that are related to malignant traits and immunotherapy, GSEA was conducted using the Hallmark and KEGG pathway gene sets. We noted that EMT, unfolded protein response, TNFa-signaling via NF-κB, DNA repair, oxidative phosphorylation, glycolysis, G2M checkpoint, and hypoxia were significantly enriched in high-risk patients, which were also validated in KEGG pathways analysis. These pathways have been associated with responsiveness or resistance to ICB therapy (39, 79, 80), suggesting the relevance of the signature to the biology of tumor progression and immunotherapy.

The role of the signature gene MOGAT2 in the tumorigenesis of NSCLC remains unclear, in contrast to other well-studied genes. The Acyl-CoA: monoacylglycerolacyltransferase (MGAT) family has three members (MOGAT1, -2, and -3) that catalyze the first step in TAG production, conversion of monoacylglycerol (MAG) to diacylglycerol (DAG). High triacylglycerol (TAG) levels correlate with metabolic syndrome severity (81). MOGAT enzymes convert monoacylglycerol (MAG) to diacylglycerol (DAG), which is then converted to TAG by DGAT. MOGAT2 inhibition shows therapeutic benefits in mice (81), improving energy expenditure and restoring normal fat absorption. This might suggest that MOGAT2 deficiency could increase energy to promote lung cancer growth, while this need to be further investigated.

Using CRISPR/Cas9 technology, we deleted MOGAT2 in H1299 cells and observed that its significant deficiency increased the proliferative potential and migratory capability of these LUAD cells, as demonstrated by CCK8 and transwell assays. Cell cycle analysis indicated that MOGAT2 knockdown promoted G2 phase arrest, aligning with the enrichment of the G2M checkpoint pathway in high-risk patients according to GSEA analysis. Some studies offer valuable insights. For instance, previous research has shown that MOGAT2 deficiency enhances colorectal carcinoma growth by activating the NF-κB pathway. NF-κB is known to play a crucial role in cell proliferation and apoptosis and is a therapeutic target in lung cancer (82). Additionally, NF-κB negatively regulates GADD45, a checkpoint protein that controls the G2/M phase transition of the cell cycle (83). Therefore, exploring the effect of MOGAT2 deficiency on GADD45 expression could be a promising approach. This study is the first to suggest that low MOGAT2 expression may promote LUAD growth (56). Furthermore, we found that MOGAT2 expression positively correlates with immune and TME scores, as well as with B cells, CD4^+^ T cells, myeloid dendritic cells, and neutrophils, suggesting its significant role in the TME. Further *in vivo* and *in vitro* validation of these findings are warranted.

The robustness of the signature showed robust performance in multiple cohorts and could be an independent predictor for LUAD, while several limitations should be taken into consideration when interpreting the signature. The retrospective nature of the signature requires multi-centered large cohorts’ validation. Using CRISPR/Cas9 technology, we knocked down MOGAT2 in lung cancer cells. We observed that all isoforms of MOGAT2 were affected to varying degrees. This suggests that the sgRNA may influence protein stability, potentially through mechanisms such as reduced translation, increased degradation, or others. Further experiments are essential to elucidate these effects. We observed that low MOGAT2 levels correlated with adverse clinical outcomes and enhanced cell proliferation and migratory capabilities. Further investigation into its molecular mechanisms in LUAD progression is essential, including studying the effects of MOGAT2 overexpression on cell proliferation and MOGAT2 deficiency on modulating the tumor microenvironment in both *in vitro* and *in vivo* models. The signature revealed the different TIME phenotypes of LUAD and predicted immunotherapy effectiveness, the correlations of these genes in mediating tumor niche that was related to therapeutics need investigation.

## 5 Conclusions

In summary, we presented a FAMG-based prognostic risk-stratification for reflecting TIME and stratifying the benefits of immunotherapy. In addition, low MOGAT2 correlated with worse outcomes and promote cell proliferation, and migratory potential. Further verification in clinical settings is underway by collaborating with hospitals and explorations *in vivo* may shed light on the role of FAMGs in LUAD and enable personalized immunotherapy.

## Data Availability

The original contributions presented in the study are included in the article/**Supplementary Material**. Further inquiries can be directed to the corresponding author/s.
