# Supplementary material for: Fatty acid metabolism prognostic signature predicts tumor immune microenvironment and immunotherapy, and identifies tumorigenic role of MOGAT2 in lung adenocarcinoma
Source: Front Immunol. 2024 Oct 16;15:1456719. doi: 10.3389/fimmu.2024.1456719 (PMC11521851; doi:10.3389/fimmu.2024.1456719)
Supplement: Supplementary file 1 [file DataSheet1.pdf]

Accession #	Resource	Platform	Samples	Experiment type	PMID
TCGA_LUAD	TCGA	RAN-seq	535	Expression profiling by RNA-seq	https://xenabrowser.net/
GSE72094	GEO	GPL1504	398	Expression profiling by array	26477306
GSE11969		GPL7015	90	Expression profiling by array	16549822/21465578
GSE30219		GPL570	85	Expression profiling by array	23698379
GSE31210		GPL570	226	Expression profiling by array	22080568/23028479
GSE50081		GPL570	127	Expression profiling by array	24305008
GSE68465		GPL96	442	Expression profiling by array	18641660
