# Supplementary material for: Fatty acid metabolism prognostic signature predicts tumor immune microenvironment and immunotherapy, and identifies tumorigenic role of MOGAT2 in lung adenocarcinoma
Source: Front Immunol. 2024 Oct 16;15:1456719. doi: 10.3389/fimmu.2024.1456719 (PMC11521851; doi:10.3389/fimmu.2024.1456719)
Supplement: Supplementary file 2 [file DataSheet2.pdf]

Pathway	Enrichment Score	NES	NOM p-val	FDR q-val
HALLMARK_GLYCOLYSIS	0.68	2.2	0	0.001
HALLMARK_MTORC1_SIGNALING	0.71	2.07	0	0.003
HALLMARK_MYC_TARGETS_V1	0.77	2.05	0	0.003
HALLMARK_HYPOXIA	0.6	2	0.002	0.004
HALLMARK_EPITHELIAL_MESENCHYMAL_TRANSITION	0.63	1.92	0.008	0.008
HALLMARK_E2F_TARGETS	0.73	1.89	0	0.01
HALLMARK_MYC_TARGETS_V2	0.76	1.88	0	0.008
HALLMARK_G2M_CHECKPOINT	0.71	1.86	0.002	0.011
HALLMARK_UNFOLDED_PROTEIN_RESPONSE	0.64	1.86	0	0.01
HALLMARK_TNFA_SIGNALING_VIA_NFKB	0.57	1.79	0.026	0.022
HALLMARK_OXIDATIVE_PHOSPHORYLATION	0.67	1.79	0.02	0.022
HALLMARK_DNA_REPAIR	0.61	1.75	0.012	0.031
HALLMARK_ESTROGEN_RESPONSE_LATE	0.47	1.72	0.01	0.035
