# Supplementary figures and images for: Fatty acid metabolism prognostic signature predicts tumor immune microenvironment and immunotherapy, and identifies tumorigenic role of MOGAT2 in lung adenocarcinoma
Source: Front Immunol. 2024 Oct 16;15:1456719. doi: 10.3389/fimmu.2024.1456719 (PMC11521851; doi:10.3389/fimmu.2024.1456719)

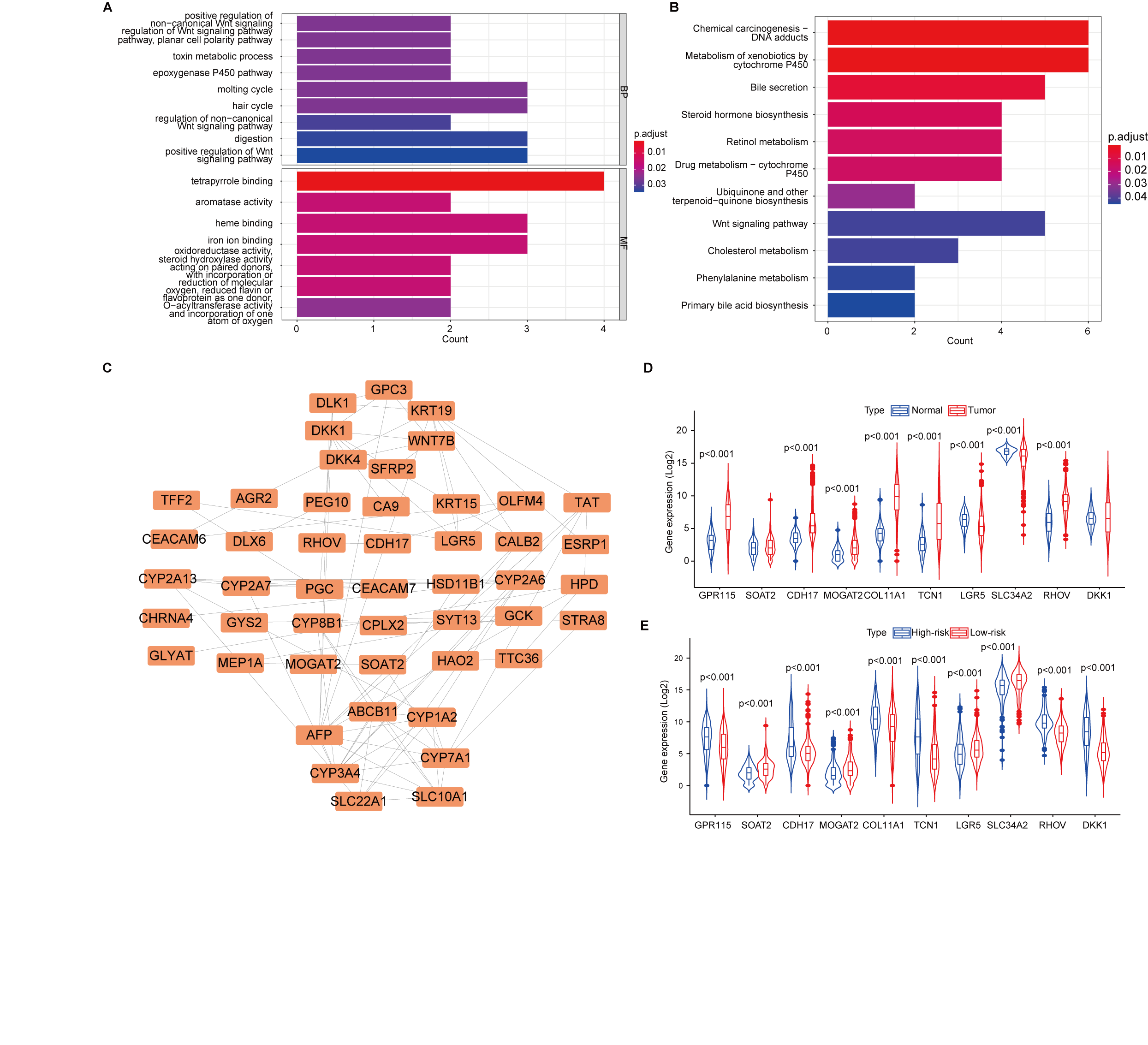

Supplement: Supplementary file 3 [file Image1.tif]

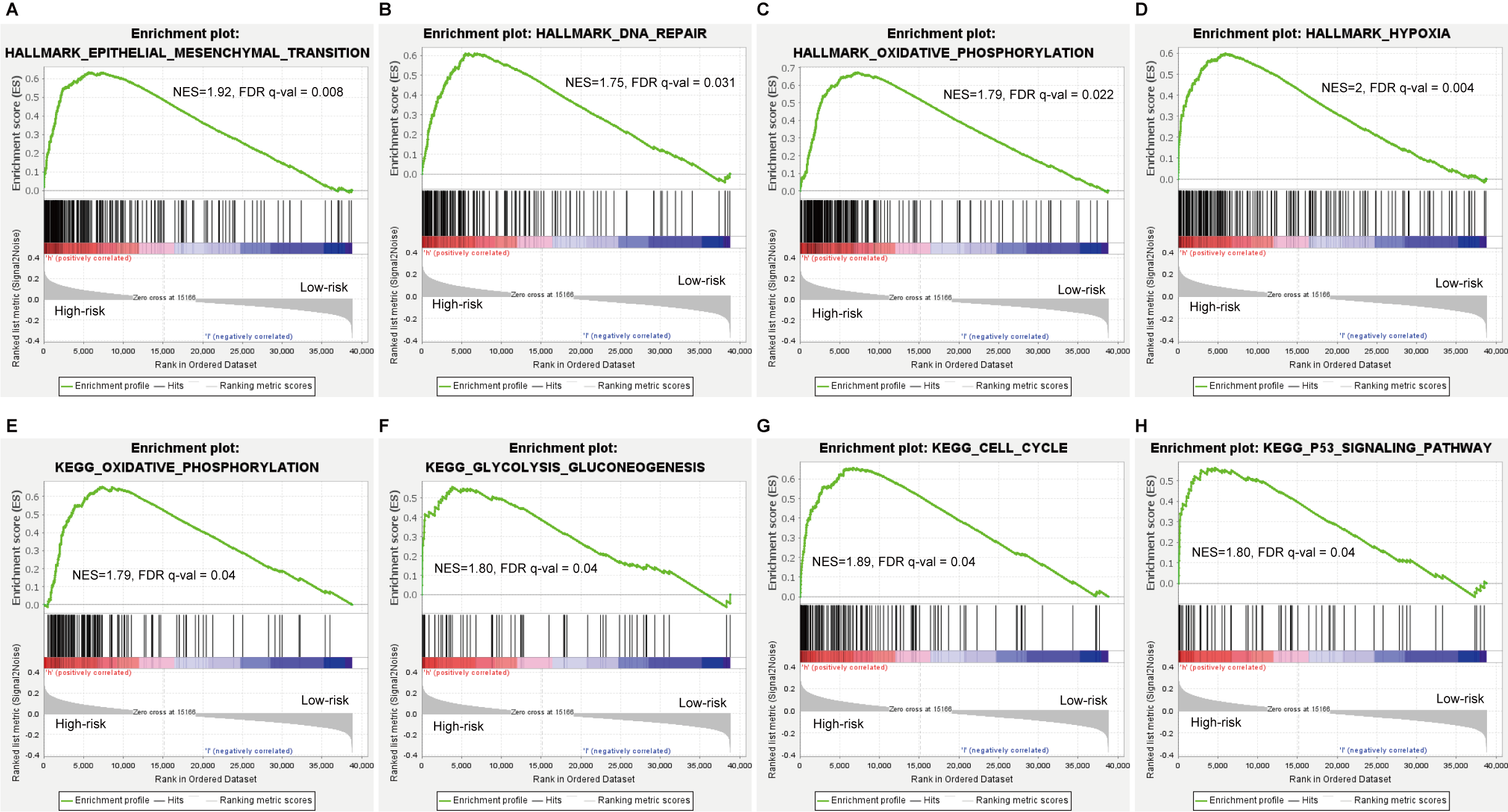

Supplement: Supplementary file 4 [file Image2.tif]

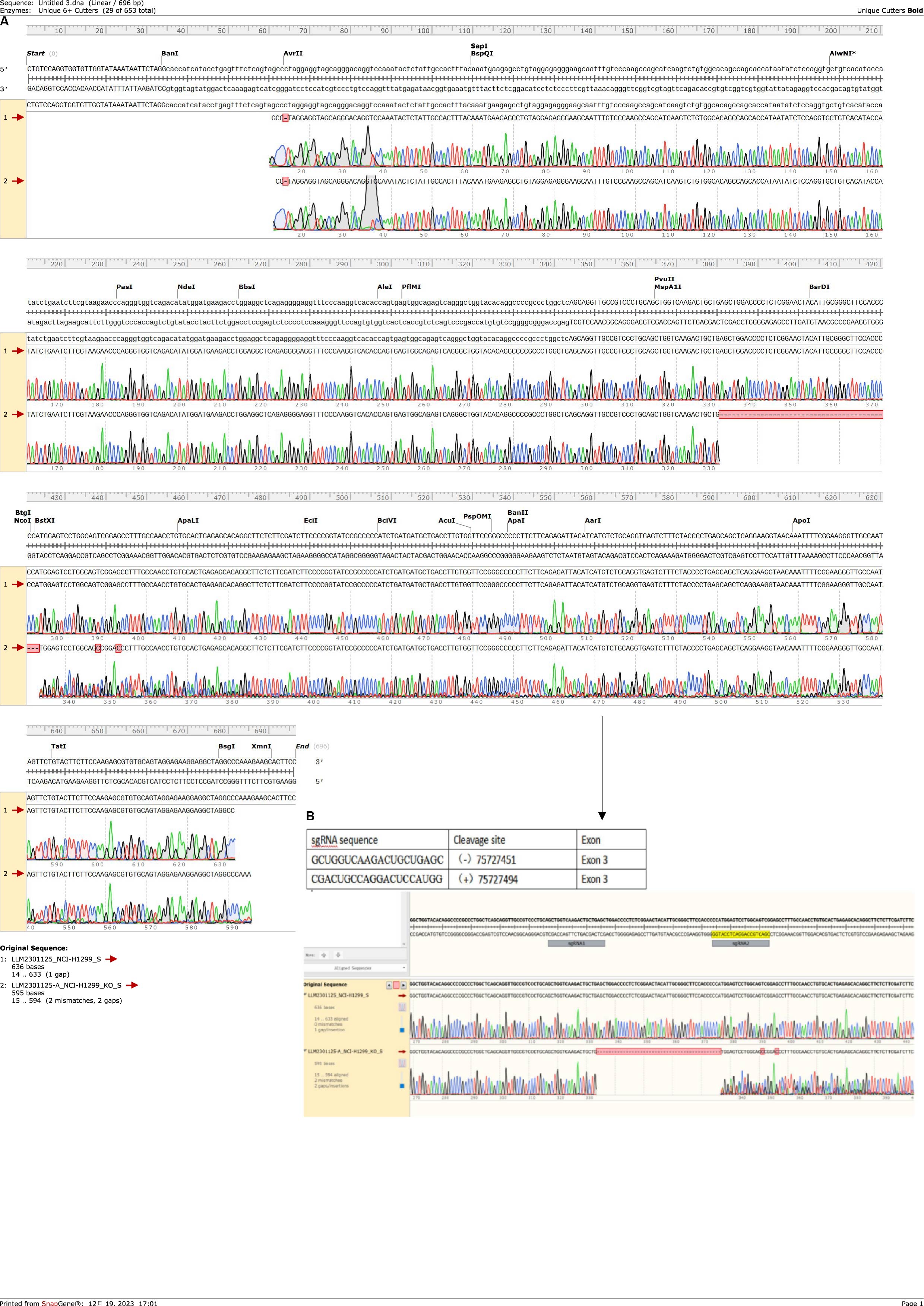

Supplement: Supplementary file 5 [file Image3.tif]

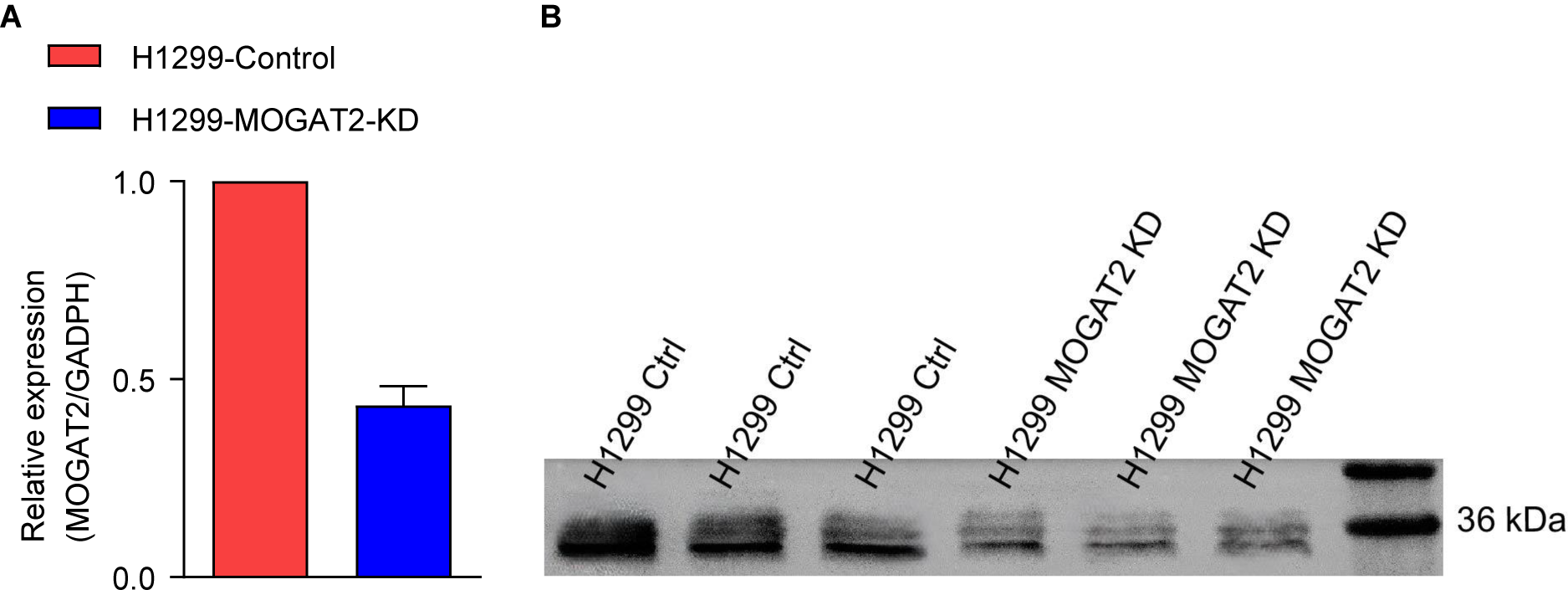

Supplement: Supplementary file 6 [file Image4.tif]

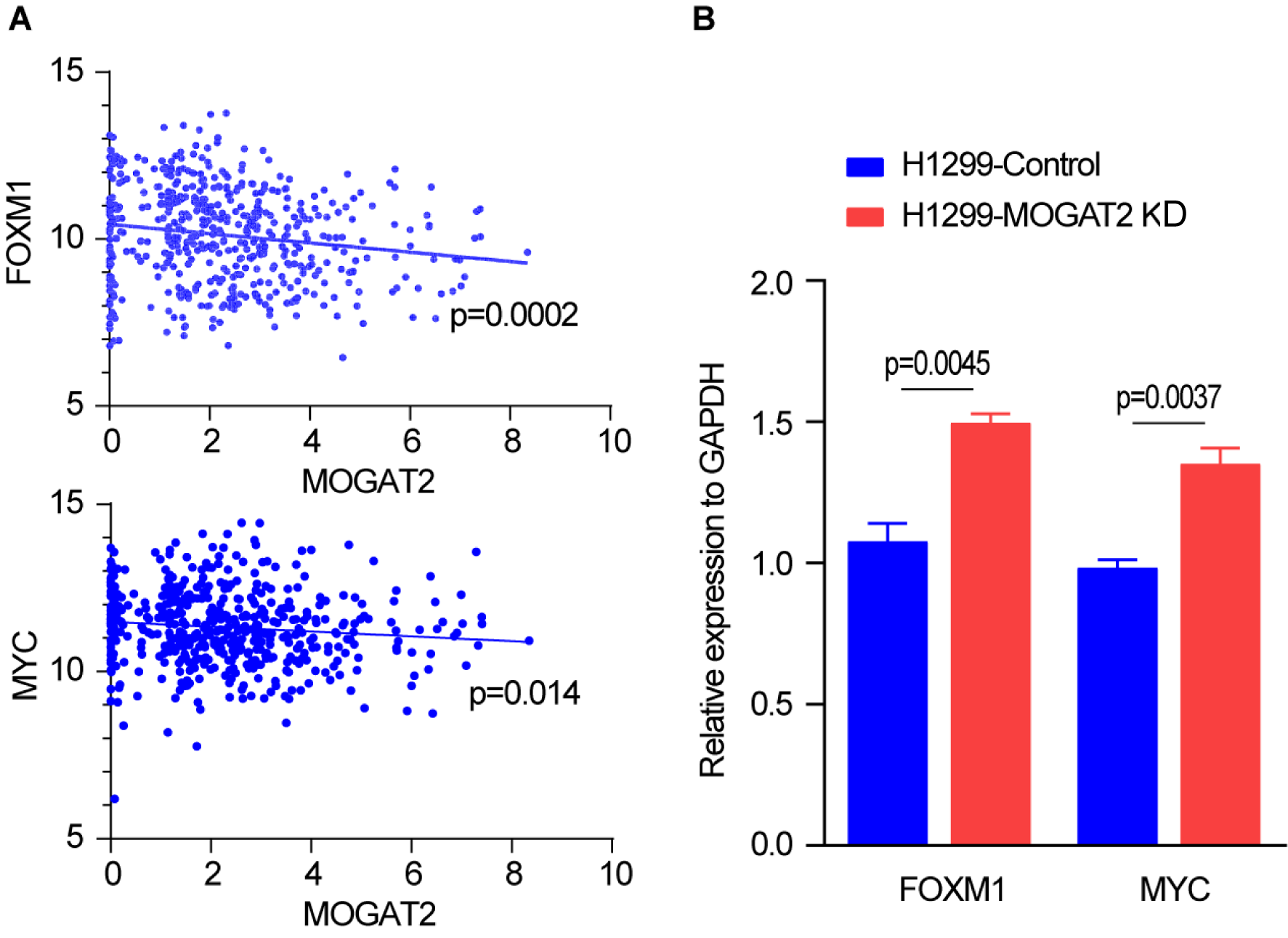

Supplement: Supplementary file 7 [file Image5.tif]

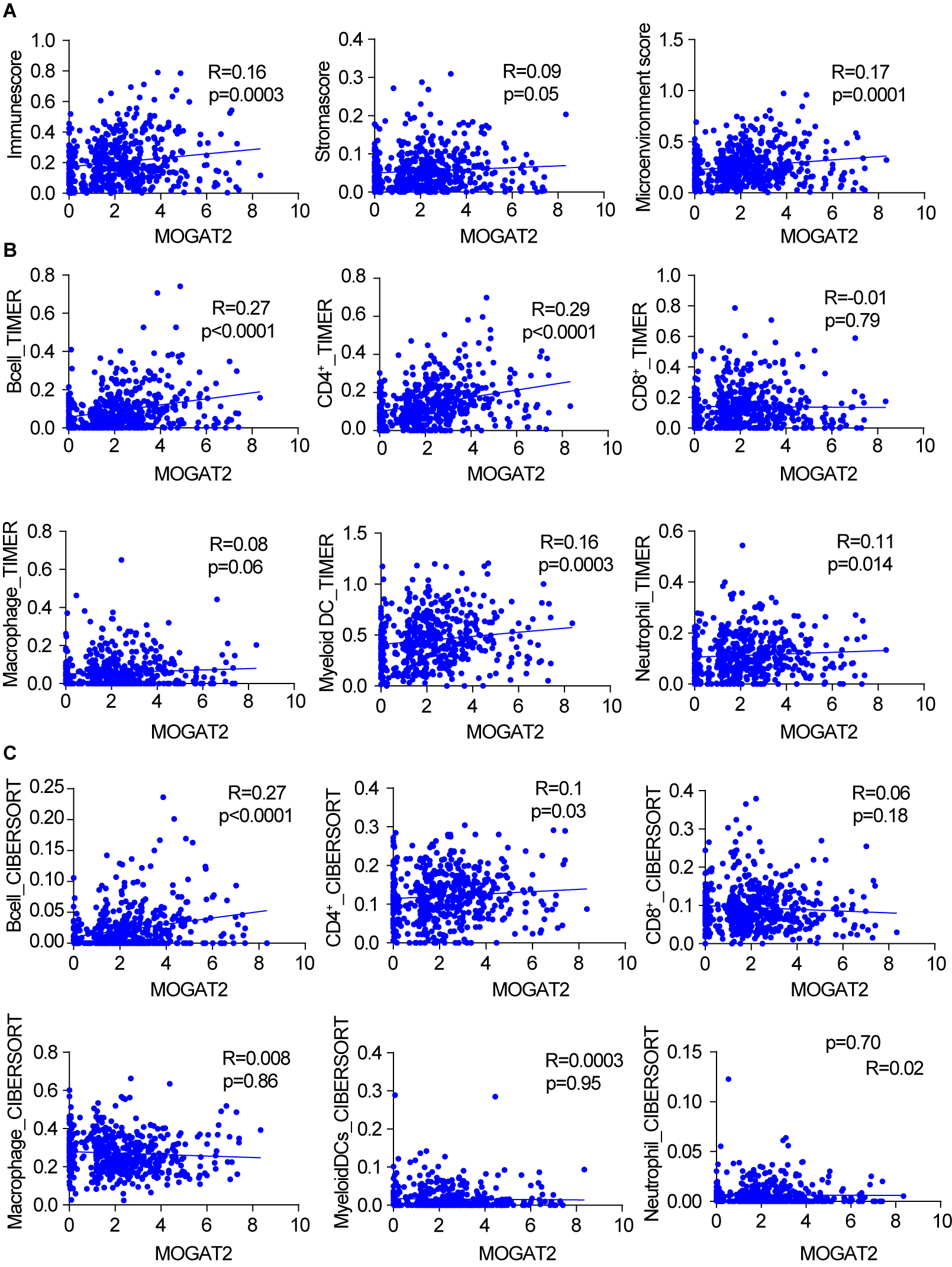

Supplement: Supplementary file 8 [file Image6.tif]
